# Modern Match Making.—How It Is Done in Paris

**Published:** 1874-04

**Authors:** 


### MODERN MATCH MAKING.—HOW IT IS DONE IN PARIS.

### "YOU TAKES YOUR CHOICE AND PAYS YOUR MONEY."

In France housekeeping has become so expensive that most men hesitate about marrying—at all events men no longer marry young in certain society. A man must have his position insured before thinking of marrying, and when that is done he is no longer of the age when men obey the impulses of the heart at the expense of the head. Of ten men who think of marrying there are nine who put the questions in the following order: Is she rich, pretty, healthy, of a good family? Then, for most men, marriage is first a money matter, next a matter of self-love, and finally of domestic peace. The other questions come after.

### MATRIMONIAL AGENTS.

Hence, men do not fear to apply to matrimonial agents directly, or get themselves recommended by a mutual friend. The agent receives his client. He studies him deeply enough to be able to say up to what point he is elegant, *distingué*, gallant and well bred, or else to what degree he is awkward, innocent, bashful, or ignorant of the habits of society. In every marriage there are two parts, that of the intermediary and that of the interested party; and the agent always succeeds better with men who have no great advantage to offer, but who made personal efforts in the cause, than with men who could do nothing by themselves. But it is not so with

### YOUNG LADIES WHO WANT HUSBANDS.

A young lady would never forgive her future husband for having married her through an agency. "What, sir," she would say to him, "you have married me, then, only for my money—through an agent? How horrid!" And the couple would be forever unhappy. Hence the agent vanishes as soon as the first formalities are accomplished. The aspirant signs a

### WRITTEN ENGAGEMENT TO PAY SIX PER CENT.

on the dowry immediately after his marriage with a person named in the treaty, and then the agent is seen no more.

### HOW IT IS WORKED.

His place, however, is then taken by intermediaries, such as many others you meet everywhere; such as men whom you elbow in all *salons*, and who are overwhelmed with tender care, because they pass for having numbers of young men to marry. The latter then goes to work. He pays a visit to the family of the young lady. He there learns, for instance, that next evening she will be at a ball. He then, by hook or by crook, procures an invitation to the ball for the aspirant. At the ball, while the inter mediary is talking with the young lady to be married, or with her mother, the aspirant comes up and bows to the intermediary, who is

CHARMED AT THE MEETING, and introduces him to the ladies. The aspirant dances with the young lady, whose name he does not know, nor that of her family, which only makes him the more natural. The intermediary a few days later becomes the interpreter of the feelings raised in the young man by this first meeting; other interviews are arranged, and if a marriage follows, neither the young lady herself nor her family ever know that it was all done by a matrimonial agency, and when the mother and the bride announces the marriage, the former never fails to observe, "Yes, dear Madam, people are quite right when they say that marriages are made in Heaven."

Sometimes, however, agents have to do with ungrateful fellows, who refuse to pay after having pocketed the dowry. These are the

THORNS IN THE TRADE, for agents must go to law, and, even if they win, the profession suffers. But often, too, they meet with delicate and grateful souls. In one of the cities of the north was an intermediary, who called an agent's attention to a young lady with three millions for her portion, who would only marry a man with a title, and who should marry her for herself and not for her money. The agent chose from the lists a Count, an engineer, without any fortune, very respectable and very cool. He got himself appointed to a post in the town in question, went there *incognito*, saw the intermediary, who showed him the young lady, then went away again to return in time for a ball given by the Prefect, to which the intermediary had got him invited. He took his stand near the door, and when the young lady entered he uttered a cry of admiration, so sincere, so *naïf*, that she herself started, and three months after he married her. "Well, sir," said the agent, subsequently, "he not only paid me the sum agreed upon, but ever since he has been most obliging. As for the young Countess, she is as happy as she was the first day, and quite as convinced as on the first day that her husband did not know he was falling in love with a dowry of three millions when he saw her."

